# The relationship of speech intelligibility with hearing sensitivity, cognition, and perceived hearing difficulties varies for different speech perception tests

**DOI:** 10.3389/fpsyg.2015.00782

**Published:** 2015-06-16

**Authors:** Antje Heinrich, Helen Henshaw, Melanie A. Ferguson

**Affiliations:** ^1^Medical Research Council Institute of Hearing ResearchNottingham, UK; ^2^National Institute for Health Research–Nottingham Hearing Biomedical Research Unit, Otology and Hearing Group, Division of Clinical Neuroscience, School of Medicine, University of NottinghamNottingham, UK; ^3^Nottingham University Hospitals NHS TrustNottingham, UK

**Keywords:** speech perception, cognition, self-report, communication, health-related quality of life, non-verbal intelligence

## Abstract

Listeners vary in their ability to understand speech in noisy environments. Hearing sensitivity, as measured by pure-tone audiometry, can only partly explain these results, and cognition has emerged as another key concept. Although cognition relates to speech perception, the exact nature of the relationship remains to be fully understood. This study investigates how different aspects of cognition, particularly working memory and attention, relate to speech intelligibility for various tests. Perceptual accuracy of speech perception represents just one aspect of functioning in a listening environment. Activity and participation limits imposed by hearing loss, in addition to the demands of a listening environment, are also important and may be better captured by self-report questionnaires. Understanding how speech perception relates to self-reported aspects of listening forms the second focus of the study. Forty-four listeners aged between 50 and 74 years with mild sensorineural hearing loss were tested on speech perception tests differing in complexity from low (phoneme discrimination in quiet), to medium (digit triplet perception in speech-shaped noise) to high (sentence perception in modulated noise); cognitive tests of attention, memory, and non-verbal intelligence quotient; and self-report questionnaires of general health-related and hearing-specific quality of life. Hearing sensitivity and cognition related to intelligibility differently depending on the speech test: neither was important for phoneme discrimination, hearing sensitivity alone was important for digit triplet perception, and hearing and cognition together played a role in sentence perception. Self-reported aspects of auditory functioning were correlated with speech intelligibility to different degrees, with digit triplets in noise showing the richest pattern. The results suggest that intelligibility tests can vary in their auditory and cognitive demands and their sensitivity to the challenges that auditory environments pose on functioning.

## Introduction

One of the overarching aims of audiological (re)habilitation is to improve communication skills and participation in everyday life by reducing activity limitations and participation restrictions (e.g., Boothroyd, 2007) The success of any intervention, such as hearing aid fitting, can be assessed using different aspects of communication such as behavioral measures of speech perception, or subjective questionnaires of self-reported hearing-related, or generic health-related quality of life (HRQoL). One way of conceptualizing communication and how to measure it, is by placing it within the World Health Organization’s International Classification of Functioning, Disability and Health (ICF: WHO, 2001). The ICF framework suggests that an individual’s level of functioning is not simply the consequence of an underlying health condition but instead should be thought of as a multifactorial concept that includes a person’s body *functions and structures*, the *activities* they perform and the social situations they *participate* in. All of these factors can be subject to external environmental and internal personal influences (Stucki and Grimby, 2004). Conceptualizing hearing, listening, and communication within this framework places hearing loss as a body *function*, listening (e.g., to speech in noise) as *activity*, and communication as *participation* (e.g., Saunders et al., 2005; Hickson and Scarinci, 2007; Danermark et al., 2010). Experimentally it has been shown that while hearing sensitivity affects listening in a variety of situations (Humes and Roberts, 1990; van Rooij and Plomp, 1990) it has also become increasingly clear that hearing loss alone cannot account for speech perception difficulties, particularly in noise (Schneider and Pichora-Fuller, 2000; Wingfield and Tun, 2007). As a consequence, the role of cognition for speech perception has come under scrutiny. Research so far has led to the general agreement that a relationship between cognition and speech-in-noise (SiN) perception exists but the nature and extent of the relationship is less clear. No single cognitive component has emerged as being important for all listening situations, although working memory (WM), specifically as tested by reading span, appears to be important in many situations (for a review, see Akeroyd, 2008).

Crucially, WM has no universally accepted definition. One definition that is widely used particularly in connection with speech perception, posits that WM capacity refers to the ability to simultaneously store and process task-relevant information (Daneman and Carpenter, 1980). Tasks have been designed that differ in the emphasis they put on storage and processing components. An example of a task with an emphasis on the storage component is the Digit Span forward task (Wechsler, 1997), an example of a task that maximizes the processing component is the Reading Span task (Daneman and Carpenter, 1980). Tasks that put a more equal emphasis on both storage and processing aspects are the Digit Span backward and the visual letter monitoring (VLM) task. WM is often correlated with speech perception, particularly when the speech is presented in multi-talker or fluctuating noise. Moreover, this correlation is often larger when the WM task contains a large processing component (Akeroyd, 2008). However, despite these general trends results have been less clear-cut. For instance, some (Desjardins and Doherty, 2013) but not all (Koelewijn et al., 2012) studies showed the expected significant correlation between reading span and SiN perception. In addition, some studies showed significant correlations between SiN perception and forward and backward digit span (Humes et al., 2006), and VLM (Rudner et al., 2008) even though these WM tasks do not maximize the processing component.

Defining WM in terms of storage and processing capability is not the only option. Other definitions of WM emphasize the role of inhibition of irrelevant information (Engle and Kane, 2003), resource-sharing, the ability to divide and switch attention (Barrouillet et al., 2004), and memory updating (Miyake et al., 2000). Importantly, these have also been linked to SiN perception (e.g., Schneider et al., 2010; Mattys et al., 2012). Finally, it is important to note that the recent focus on cognitive contributions does not imply that hearing sensitivity is not important. An approach that considers the interactive effect of both like the current study is most likely to advance our understanding of speech in noise difficulties (Humes et al., 2013).

Another factor that adds complexity to the relationship between speech perception and cognition is the type of speech perception test used. Two aspects important in a speech perception test are the complexity of the target speech and the complexity of the background noise. The target speech can vary from single phonemes to single words to complex sentences, while the background noise can vary from a quiet background to steady-state noise to a highly modulated and linguistically meaningful multi-talker babble. As a result, the same cognitive test can correlate significantly with speech perception when using a more complex sentence perception test (Desjardins and Doherty, 2013; Moradi et al., 2014) but not when using less complex syllables (Kempe et al., 2012). Similarly, correlations with cognitive processes are greater when listening to speech in adverse noisy conditions than when listening in quiet (e.g., van Rooij and Plomp, 1990; Wingfield et al., 2005; Rönnberg et al., 2010). In order to cover a wide range of listening situations with relatively few speech perception tests we varied the complexity of both the target and background signal simultaneously. In the low complexity condition listeners were required to discriminate phonemes in quiet, in the medium condition to recognize words in a steady-state background noise and in the most complex condition to comprehend sentences presented in a modulated noise.

When speech perception is measured in noise, the signal-to-noise ratio (SNR) can be manipulated in one of two ways. First, the noise level is fixed and the signal level of the target is varied, or second, the level of the target is fixed and the level of the noise varied. Both methods of setting SNR are used in speech research (Mayo et al., 1997; Smits et al., 2004, 2013; Vlaming et al., 2011), usually without any discussion on how this methodological variation may affect speech perception. Conversely, in audiology practice, the preferred method for changing SNR is to fix the noise and decrease the signal levels (Wilson et al., 2007), because there is an understanding that increasing the noise level can add a quality of annoyance to the signal that is unrelated to intelligibility (Nabelek et al., 1991). Using the Digit Triplet Test, we explored the consequences of both methods of adjusting the SNR for speech perception and their relationships with cognitive function and self-report measures.

Self-report questionnaires assess subjective experience. A recent systematic review identified 51 different questionnaires that were used by studies that met the review’s specific research requirements (Granberg et al., 2014). Questionnaires can be considered as assessing either generic HRQoL or disease-specific (e.g., hearing) aspects (Chisolm et al., 2007). One example of a generic and widely used HRQoL questionnaire is the EQ-5D (The EuroQol Group, 1990). It assesses an individual’s ability to perform activities and measures the resulting limits on levels of participation. However, it has been shown to be insensitive to hearing loss (Chisolm et al., 2007; Grutters et al., 2007). Therefore, an additional set of questions based on the same assessment principles have been developed that extends the EQ-5D and is sensitive to hearing-specific health states such as communication, self-confidence, and family activities (Arlinger et al., 2008). Alternatively, hearing-specific questionnaires can measure activity limitations and participation restrictions, with different questionnaires assessing different aspects of listening. For example, the Auditory Lifestyle and Demand Questionnaire (ALDQ; Gatehouse et al., 1999) assesses listening situations and demands in terms of frequency and importance, the Speech, Spatial, and Qualities of Hearing Questionnaire (SSQ; Gatehouse and Noble, 2004) assesses the listener’s ability to perform in particular listening situations, and the Glasgow Hearing Aid Benefit Profile (GHABP; Gatehouse, 1999) assesses activity limitations and participation restrictions associated with listening to speech. However, relatively little is understood about the relationship between different listening situations as measured by hearing-specific questionnaires and performance on various speech perception tests (Cox and Alexander, 1992; Humes et al., 2001).

In addition to examining the relationship between self-report and speech perception in general, we also investigated whether the procedural differences for varying SNRs affect the relationship between speech perception and self-report scores. If for instance setting the SNR by changing the level of noise rather than the signal leads to increased noise levels (as would occur if the SNR for 50% performance threshold is negative), the resulting SNR may become uniquely associated with self-report scales on auditory functioning in noisy environments.

In summary, the current study aimed to assess the relationship between (1) speech perception and cognition, and (2) speech perception and self-report, and how these relationships changed when speech perception tests differed in complexity. Based on previous research we made the following predictions:

Aim 1: Assessing the relationship between speech perception and cognition

(1.1) Speech perception performance will be associated with cognition, and this will be moderated by hearing sensitivity.
(1.2) The contribution of cognition will increase as the complexity of the speech perception task increases.
(1.3) Where procedural differences in identifying SNR occur while the speech and background signals are identical, we expect comparable associations with cognition if these associations are driven by signal complexities and not procedural differences.

Aim 2: Assessing the relationship between speech perception performance and self-reported outcomes

(2.1) Hearing-specific questionnaires will demonstrate a greater association with speech perception performance than generic health measures.
(2.2) Correlations with speech perception performance will be largest for questionnaires that capture aspects of listening important for that particular speech perception test.
(2.3) Procedural differences in identifying SNR for speech perception performance may lead to different associations with self-report scales. In particular, increasing the level of background noise to reduce perceptual accuracy may be uniquely associated with functioning in challenging auditory environments.

By better understanding the relationship between behavioral and subjective measures of listening, this study aims to enable healthcare practitioners and researchers to be more informed in their choice of the outcome measures (either speech perception tests or questionnaires) that relates explicitly to the needs and goals of a particular individual (Gatehouse, 2003) or research question.

## Materials and Methods

The data were a subset of a randomized controlled trial to assess the benefits of a home-delivered auditory training program (Ferguson et al., 2014) in which 44 adults with mild sensorineural hearing loss (SNHL) completed outcome measures of speech perception, cognition, and self-report of health and hearing ability. Here, we only examine the baseline data from the participants’ initial visit. The study was approved by the Nottingham Research Ethics Committee and Nottingham University Hospitals NHS Trust Research and Development. Signed, informed consent was obtained.

### Participants

Participants (29 male, 15 female) were aged 50–74 years old (mean = 65.3 years, SD = 5.7 years) with mild, symmetrical SNHL (mean hearing thresholds averaged across 0.5, 1, 2, and 4 kHz = 32.5 dB HL, SD = 6.0 dB HL, with a left–right difference of <15 dB). All participants spoke English as their first language, and were paid a nominal attendance fee and travel expenses for their visit.

### Procedure

Audiometric measurements (middle-ear function and pure-tone air-conduction thresholds) were obtained in a sound-attenuated booth. All other testing (cognitive tests, speech perception tests and self-report questionnaires) took place in a purpose-designed quiet test room. Outcome measures were administered in the same order for all participants.

### Outcome Measures

#### Audiological

Outer and middle ear functions were checked by otoscopy and standard clinical tympanometry using a GSI Tympstar (Grason-Stadler, Eden Prairie, MN, USA). *Pure-tone air conduction thresholds* (0.25, 0.5, 1, 2, 3, 4, 8 kHz) were obtained for each ear, following the procedure recommended by the British Society of Audiology (British Society of Audiology, 2011), using a Siemens (Crawley, West Sussex, UK) Unity PC audiometer, Sennheiser (Hannover, Germany) HDA-200 headphones, and a B71 Radioear (New Eagle, PA, USA) transducer in a sound-attenuating booth. The better-ear-average (BEA) across octave frequencies 0.5–4 kHz was derived and is reported here.

#### Cognitive

The *Matrix Reasoning* subtest of the Wechsler Abbreviated Scale of Intelligence (WASI; Wechsler, 1999) estimated the non-verbal intelligence quotient (NVIQ). The *Digit Span* (forward, then backward) from the Wechsler Adult Intelligence Scale (WAIS) Third Edition (Wechsler, 1997) was used to estimate auditory WM capacity. Pairs of pre-recorded spoken digit (0–9) sequences were presented at 70 dBA via Sennheiser HD-25 headphones. On successful recall, the sequence was increased by one digit. The test was discontinued when both sequence pairs were incorrectly recalled.

The Visual Letter Monitoring test (VLM) assessed visual WM (Gatehouse, 2003). Ten consonant-vowel-consonant (CVC) words were embedded within an 80-letter sequence displayed sequentially on a computer screen. Participants pressed the keyboard space bar when three consecutive letters formed a recognized CVC word (e.g., M-A-T). The test consisted of two runs, initially with a presentation rate of one letter/2 s, followed by one letter/1 s. Here, only responses to the faster presentation sequence were analyzed in terms of hits (accuracy in %) and reaction time (processing speed in ms).

Two subtests of the *Test of Everyday Attention* (TEA; Robertson et al., 1994) assessed focused and divided attention. In the Telephone Search (Subtest 6, focused attention) participants had to identify 20 designated key symbols, as fast as possible, and ignore all other symbols while searching entries in a simulated classified telephone directory. The score was calculated as a quotient between the total time taken to complete the test divided by the number of symbols detected. The maximum number was 20 and lower values represent superior performance. Divided attention was measured with the Telephone Search (Subtest 7, dual task) that was identical to subtest 6 except that participants had to count a string of 1-kHz tones while searching the directory. The task score was considered separately, and in conjunction with subtext 6 (dual task decrement, DTD). For statistical analyses the scales for both tests were reversed to harmonize the direction of scoring for all cognitive tests with higher scores indicating a better performance in all instances.

#### Speech Perception

The *Phoneme Discrimination* test measured the discrimination threshold for one phoneme continuum (/a/ to /e/) with 96 steps. Stimuli were delivered through Sennheiser HD-25 headphones at a fixed level of 75 dBA. A three-interval, three-alternative forced choice, oddball paradigm using a step size of 2 combined with a three-down, one-up staircase procedure starting with the second reversal was used to determine the 79% correct point on the psychometric function (Levitt, 1971). Feedback was given. Phoneme discrimination threshold (PD; %) was the average of the last two reversals over 30 trials.

The *Digit Triplet Test* (Smits et al., 2004; Smits and Houtgast, 2005) presented series of three digits against a steady, speech-shaped background noise. Six lists of digits were randomized to minimize order effects. The 50% threshold for digits perception was determined in two ways, (i) the speech level was fixed at 65 dB SPL and the noise level was adaptively varied (DTT_V N_) (ii) the noise level was fixed at 65 dB SPL and the speech level was adaptively varied (DTT_V S_). Both noise and speech varied in 2 dB steps in a one-down, one-up paradigm for 27 trials starting with a SNR of +5 dB.

The *Adaptive Sentence List* (ASL; MacLeod and Summerfield, 1990) comprised 30 sentences presented in a 8-Hz modulated noise. Sentences consisted of five words, including three key words (e.g., The lunch was very early), which all needed to be correctly repeated for a sentence to be scored as correct. In keeping with current audiological practice, the noise level was fixed at 60 dBA, and the speech level was adaptively varied first in 10 and 5 dB steps in a one-up, one-down procedure for the first two reversals changing to a three-down, one-up paradigm, and a 2.5 dB step size starting with a SNR of +20 dB. The speech reception threshold was the average SNR of the last two reversals.

All speech perception in noise tests were presented in free-field at a distance of 1 meter. In all speech perception tests a lower score indicates a better performance.

#### Self-Report of Health-Related Quality of Life (HRQoL)

*EQ-5D* (The EuroQol Group, 1990) is a standardized generic self-report questionnaire measuring HRQoL. It comprises five questions, each on a three-point scale (no problems, some problems, extreme problems) that assess general life quality as it relates to mobility, self-care, usual activities, pain/discomfort, and affective disorders (depression/anxiety; general EQ-5D). In addition, a set of questions focusing on hearing-specific health states (hearing-specific EQ-5D) was used to assess aspects of life directly related to hearing loss, such as communication, confidence, family activities, social and work activities, and energy level (Arlinger et al., 2008).

#### Self-Report of Hearing

The ALDQ (Gatehouse et al., 1999) measures frequency and impact of hearing loss by inquiring about a variety of listening situations (*n* = 24). Both dimensions are evaluated on a three-point scale (Frequency: very rarely/sometimes/often; Importance: very little/some importance/very important). Questions range from listening to sounds of various intensities, to listening to distorted or masked speech to listening to various sound types. Here, an average of both subscales is used where a higher value indicates a richer auditory environment of higher importance to the listener.

The GHABP (Gatehouse, 1999) assesses activity limitations and participation restrictions using four predefined situations (e.g., TV level set to suit other people, conversation with one other person in no background noise, in a busy street, with several people in a group) on a five-point scale (1 = no difficulty to 5 = cannot manage at all). The mean scores for the two subscales of activity limitations and participation restrictions were converted to a percentage and then averaged for an overall score of communication ability.

The SSQ (Gatehouse and Noble, 2004) assesses abilities and experiences of hearing in difficult listening situations. It comprises 49 questions across a variety of hearing domains such as speech perception in a variety of competing contexts (Speech, n = 14), using directional, distance, and movement components to hear (Spatial, *n* = 17) and judging quality of hearing regarding clarity and ability to identify different speakers, musical pieces/instruments, and everyday sounds (Qualities, *n* = 18). Participants rate their hearing ability along a 0–10 visual analog scale for each questions (0 = not at all to 10 = perfectly). Mean scores for each subscale were calculated and averaged for an overall mean score.

Scales were reversed for all further analyses for the general EQ-5D, the hearing-specific EQ-5D, and GHABP in order to assign the highest values to scores of best functioning and richest environment.

### Data Analysis

#### Relationship with Cognitive Tests

Simple Pearson product-moment correlations between each of the four speech perception tests and age, BEA hearing thresholds, and cognitive measures were calculated. Because performance on all but one (phoneme discrimination) speech perception test was significantly correlated with hearing thresholds, partial correlations between speech perception and cognition were calculated by controlling for BEA. Differences in correlations between cognitive tests and speech perception tests were assessed by computing *z*-values for differences between correlations following Steiger (1980).

A main interest of the study was the predictive value of performance on cognitive tests for each speech perception test. However, the number of cognitive tests was fairly large (seven) for a relatively modest sample size of 44 participants. In order to reduce the number of cognitive tests (predictors) for the subsequent regression analysis, a principal component analysis (PCA) was performed in one of two ways. First, a single component solution, explaining the maximum amount of variance among all seven cognitive tests, was extracted. Second, using an orthogonal rotation with Kaiser Normalization, all components following the Kaiser criterion (KMO) of eigenvalues > 1 were extracted, which in this case resulted in a two-factor solution. Both solutions, the single-factor and the two-factor solution, were subsequently used as predictors in separate two-step forward hierarchical regression analyses in which BEA was always entered in a first step to control for hearing, and the extracted one- or two-factor solutions second. Finally, the influence of hearing and cognition for each of the speech perception tests was simultaneously compared in a canonical correlation analysis (CCA) using multivariate ANOVAs to assess whether the pattern of influence of hearing and cognition differed between the four speech perception tests.

A similar analysis plan was followed for self-report, except for the following two deviations. First, no partial correlations with the control of BEA were computed for self-report measures because hearing loss is an essential component of hearing questionnaires. Second, no principal component solutions were extracted and no regression analyses were performed as self-report measures were not conceptualized as predictors for speech perception performance.

## Results

A description of all variables is presented in **Table 1**

**Table 1:** Mean, SE, and range for all measurements.

Domain	Function	Tests		Mean	SE	Range
Listening activity		PD (%) (*n* 43)		65.35	1.43	54 to 99
		DTT (SNR)		-6.60	0.23	8.50 to 1.20
		DTT (SNR)		-6.99	0.25	9.20 to 1.00
		ASL (SNR)		1.34	0.60	5.00 to 11.25
Hearing		BEA (0.5–4 kHz; dB HL)		32.50	0.90	21.25 to 45.00
Cognition	NVIQ	Matrix Reasoning		22.14	0.79	7 to 30
	Working memory (WM)	Digit Span	Forward	9.68	0.31	7 to 14
			Backward	6.68	0.29	4 to 11
		VLM (*n* = 40)	Accuracy (Hits)	5.65	0.41	0 to 10
			Speed (RTms)	644.30	22.10	0 to 881
	Attention	TEA *(n* = 43)	Subtest 6	3.40	0.11	2.10 to 4.80
			Subtest 7	4.69	0.18	2.60 to 7.0
			DTD	1.28	0.15	0.10 to 3.70
Self-report	Health-related quality of life (HRQoL)	EQ-5D (*n* 42)	General	1.26	0.05	1 to 2
			Hearing-specific	1.31	0.05	1 to 2
	Self-report of hearing	ALDQ		2.10	0.04	1.52 to 2.61
		GHABP (%)		32.81	2.76	4.17 to 62.50
		SSQ		6.29	0.22	3.07 to 9.49

*PD, Phoneme discrimination; DTT, Digit Triplet Test with variable speech (DTT) or variable noise (DTT); ASL, Adaptive Sentence List; BEA, better ear average; NVIQ, non-verbal intelligence quotient; VLM, visual letter monitoring; RT, reaction time; TEA, Test of Everyday Attention; DTD, dual-task decrement; HRQoL, Health related quality of life; ALDQ, Auditory Lifestyle and Demand Questionnaire; GHABP, Glasgow Hearing Aid Benefit Profile; SSQ, Speech, Spatial and Qualities of Hearing. When deviant from n 44, n is noted for the particular test*.

### Aim 1: Assessing the Relationship between Speech Perception and Cognition

#### Prediction 1.1. Speech Perception Performance will be Associated with Cognition, and this will be Moderated by Hearing Sensitivity

##### Correlational analyses

**FIGURE 1:**
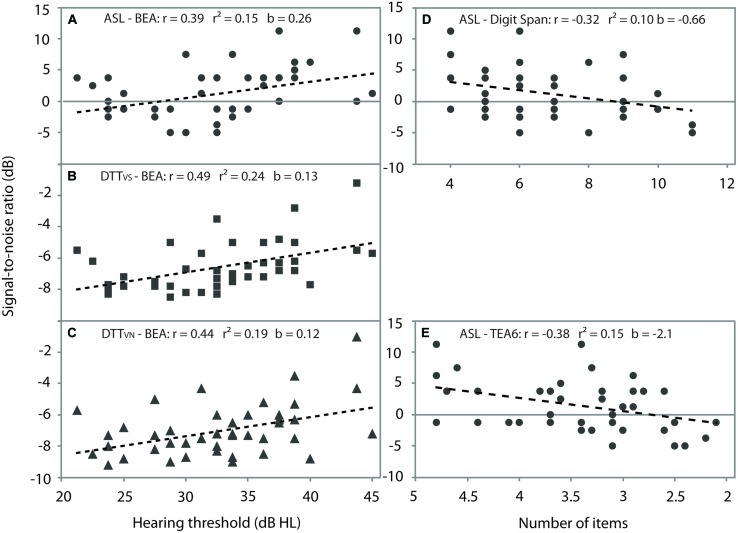
**Correlation coefficients (*r*), proportion of explained variance (*r*^2^) and predictor values (*b*) for all significant correlations**. Correlations with BEA are shown for **(A)** ASL, **(B)** DTT_V S_, **(C)** DTT_V N_, **(D)** correlations between ASL and Digit Span backward, and **(E)** ASL and TEA6. The dotted line shows the line of best fit. Acronyms as for **Table 1**

All Pearson product-moment correlations between speech perception tests, hearing thresholds and cognitive variables that were significant at *p* < 0.05 (two-tailed) are shown as scatter plots in **Figure 1** All speech perception tests except phoneme discrimination were positively correlated with BEA. Because speech perception performance was measured in SNR for a fixed intelligibility level, a lower SNR translated to better performance. The positive correlation with BEA indicated that better hearing sensitivity was associated with lower SNR values. In addition, sentence perception was negatively correlated with Digit Span backward and focused attention (TEA6) indicating that higher scores on these tasks were associated with better intelligibility. A marginal negative correlation was observed between ASL and dual attention (TEA7) indicating that better ability to divide attention was associated with better intelligibility and as a result a lower SNR. DTT_V S_ was marginally positively correlated with the DTD such that listeners showing smaller performance decrement under dual attention had lower SNRs. Neither phoneme discrimination nor DTT_V N_ were correlated with any cognitive measure. There were also no correlations between any of the speech perception tests and age.

In addition to these results, Supplementary Tables S1 and S2 report the full set of (i) bivariate correlation coefficients, and (ii) all correlations with BEA partialled out. The partial correlations led to broadly similar results as seen with simple correlations. Noteworthy were three differences. First, ASL sentence perception was now negatively correlated with NVIQ with a higher NVIQ score indicating better intelligibility and thus lower achieved SNR. Second, the previously significant negative correlation with Digit Span backward was now marginal. Third, the previously marginal positive correlation between DTT_V S_ and the DTD became significant. In summary, ASL and DTT_V S_ were associated with various tests of cognition, with a largely similar correlational pattern for bivariate and partial correlations.

In summary, in concordance with the prediction, the results show correlations between speech perception and cognitive tests, particularly in the cases of sentence perception (ASL) and DTT_V S_. Although speech perception was also correlated with hearing sensitivity, the fundamental pattern of correlation between cognition and speech did not change much when hearing loss was partialled out. This suggests a genuine role of cognition for speech perception performance.

It is also interesting to note that the significant difference between correlation coefficients is often between ASL and DTT_V S_ for a particular cognitive variable. For instance in Supplementary Table S2, a significant correlation exists between ASL and both Matrix Reasoning and TEA6. The same is not true between DTT_V S_ and Matrix Reasoning and TEA6. In addition to being significant, the correlation coefficient between these cognitive variables and ASL was also significantly larger than that between the same cognitive variables and DTT_V S_. Similarly, for TEA7, the correlation was significant with DTT_V S_ but not ASL, and the difference in correlation coefficient was in itself significant. Hence, while both DTT_V S_ and ASL correlate with cognitive measures, the correlation profile for these two speech perception tests differs, suggesting their cognitive requirements are different.

#### Prediction 1.2. The Contribution of Cognition will Increase as the Complexity of the Speech Perception Task Increases

##### Principal components analysis (PCA)

The principal component solutions based on the shared variance between all seven cognitive tests are shown in **Table 2** Extracting a single principal component explained 40% of shared variance [KMO: 0.71, Bartlett: χ^2^ (21) = 74.8, *p* < 0.0001] and showed substantial correlations with Matrix Reasoning, Digit Span forward and backward, VLM accuracy, and TEA 6 and 7 thereby representing a broad cognitive factor that includes non-verbal intelligence, WM, and attention. Only VLM Speed representing processing speed was not well represented by this latent factor.

**Table 2:** Factor loadings for all cognitive tests for the two principal component analysis.

			Single factor solution	Two-factor solution	
			General Cognition (Cogn; 40%)	Working Memory (33%)	Attention (Att; 30%)
NVIQ	Matrix Reasoning		0.74	0.41	0.67
WM	Digit Span	Forward	0.76	0.83	0.18
		Backward	0.75	0.85	0.14
	VLM	Accuracy	0.60	0.80	0.04
		Speed (RT ms)	0.04	0.28	0.41
Attention	TEA	Subtest 6	0.59	0.09	0.83
		Subtest 7	0.61	0.13	0.82

*Explained variance by each factor is in brackets. Acronyms as for **Table 1** The loadings of the cognitive tests contributing most to a particular factor are shaded*.

Alternatively, aiming for the solution with the greatest amount of explained variance by extracting all factors with eigenvalue > 1 resulted in two factors and a total explained variance of 63% [KMO: 0.71, Bartlett: χ^2^ (21) = 74.8, *p* < 0.0001)]. Factor 1, representing 33% of variance in cognitive performance, was most highly correlated with WM while Factor 2, explaining 30% of cognitive performance variance, loaded most highly on NVIQ and attention. Processing speed did not load highly on either factor. In the following, the single latent factor is referred to as General Cognition (Cogn) factor, and Factor 1 in the two-factor solution as WM factor, and Factor 2 in the two-factor solution as Attention (Att) factor.

##### Hierarchical regression analysis

Both the single Cogn factor and the two WM and Att factors were used as independent predictors in forward stepwise regression analyses on the four speech perception tests where they were always entered in a second step after hearing thresholds. The results of these analyses are reported in **Table 3** For Phoneme discrimination, neither hearing nor cognition, either as single factor or two factors contributed significantly to the performance. For the two Digit Triplet tests, only hearing made a highly significant contribution, while cognition, whether entered as one (Cogn) or two (WM, Att) latent factors, did not. For Sentence perception, both hearing and cognition made significant contributions. Intriguingly, when the two latent cognitive factors WM and Att were entered separately into the model (M2), only Att made a significant contribution to Sentence perception suggesting that it was the attentional component in the cognitive tasks that drove the link with performance for this speech perception test.

**Table 3:** Results for two forward stepwise regression models carried out for each speech perception test.

			*R*	*R*	adj *R*	SE	*R* change	*F* change	*df*1	*df*2	Significance *f* change
PD	M1&M2	BEA	0.29	0.08	0.06	9.51	0.08	3.22	1	36	0.08
	M1	Cogn	0.32	0.10	0.05	9.55	0.02	0.72	1	35	0.40
	M2	WM	0.33	0.11	0.06	9.52	0.02	0.96	1	35	0.33
		Att	0.33	0.11	0.03	9.66	0.001	0.02	1	34	0.90
DTT	M1&M2	BEA	0.49	0.24	0.22	1.43	0.24	11.76	1	37	0.002
	M1	Cogn	0.51	0.26	0.21	1.44	0.01	0.66	1	36	0.42
	M2	WM	0.50	0.25	0.21	1.45	0.01	0.25	1	36	0.62
		Att	0.51	0.26	0.19	1.46	0.01	0.44	1	35	0.51
DTT	M1&M2	BEA	0.47	0.22	0.20	1.53	0.22	10.7	1	37	0.002
	M1	Cogn	0.48	0.23	0.19	1.54	0.006	0.28	1	36	0.60
	M2	WM	0.48	0.23	0.18	1.55	0.002	0.08	1	36	0.78
		Att	0.48	0.23	0.17	1.56	0.005	0.24	1	35	0.63
ASL	M1&M2	BEA	0.39	0.15	0.13	3.84	0.15	6.64	1	37	0.01
	M1	Cogn	0.51	0.27	0.22	3.63	0.11	5.51	1	36	0.02
	M2	WM	0.42	0.18	0.13	3.84	0.03	1.12	1	36	0.30
		Att	0.54	0.29	0.23	3.61	0.12	5.68	1	35	0.02

*In all models hearing was entered first and cognition second, therefore results for hearing were identical regardless of how cognition was entered and is only reported once (M1&M2). In Model 1 (M1) for each speech perception test cognitive performance was entered as a single factor (Cogn). In Model 2 (M2) cognitive performance was entered as two separate factors representing WM and Attention. Acronyms as for **Table 1** Significant results (p < 0.05) are shaded*.

This result extends the correlational results and suggests different predictive patterns of hearing and cognition for the speech perception tests. Specifically, it shows that the role of cognition was only predictive for performance differences in sentence perception. The main limitation of this approach is that the four speech perception tests are examined in separate statistical models. CCA examines whether there are correlations between two sets of variables and checks how many dimensions are shared between them. In this case hearing and cognition comprised one set, and the four speech perception tests the other set.

##### Canonical correlational analyses

The two sets that were compared comprised hearing, represented by BEA, and cognition, represented by the single PCA factor solution (Cogn), in Set 1 and the four speech perception tests in Set 2. The overall multivariate model, based on 38 cases, indicates that there is evidence for an overall relationship between the two sets of variables (Wilks’ lambda, *p* = 0.05). Univariate regression analyses within the CCA model replicate the earlier hierarchical regression analyses by showing that performance on the DTT_V S_ [*F*(2,35) = 6.04, *p* = 0.006], DTT_V N_ [*F*(2,35) = 5.12, *p* = 0.01], and ASL [*F*(2,35) = 6.12, *p* = 0.005], but not on Phoneme discrimination [*F*(2,35) = 1.95, *p* = 0.16], showed significant contributions of at least one of the two predictor variables hearing and cognition. For the two digit triplet tests these contributions were due to hearing only (*p* = 0.03), whereas for sentence perception, both hearing (*p* = 0.027) and cognition (*p* = 0.027) contributed. The first canonical root explained 31% of shared variance, the second 9%, however, only the first root was significant (both canonical roots included: *F*(8,64) = 2.11, *p* = 0.05; first canonical root removed: *F*(3,33) = 1.10, *p* = 0.36). The correlations and canonical coefficients (loadings) for both solutions are included in Supplementary Table S3. Examination of the loadings suggests that hearing contributes about twice as much to the first root as cognition, and that the contribution of hearing and cognition were in opposite directions for the second root. Sentence perception was more affected by both root solutions than the other three speech perception tests.

In summary, based on all the statistical testing, a converging picture emerges in which cognitive tests differ in the extent to which they correlate with speech perception tests that vary in complexity. When cognition together with hearing, is considered as a predictor for speech perception performance, it only has a significant effect for sentence perception. This is true whether it is modeled as a unified variable or as a variable with subcomponents for WM and attention. Moreover, it is the attentional component of cognition that is crucial. Lastly, while the direct comparison of hearing and cognition for all four speech perception tests was limited by the small number of cases, and thus any results can only indicate tendencies, the CCA showed that the best root solution comprised both contributions from hearing and cognition and that this root was most important for modeling performance on the sentence perception test (ASL).

#### Prediction 1.3. Where Procedural Differences in Identifying SNR Occur while the Speech and Background Signals are Identical, We Expect Comparable Associations with Cognition if these Associations are Driven by Signal Complexities and not Procedural Differences

Supplementary Tables S1–S3 and **Table 3** suggest very similar results for DTT_V S_ and DTT_V N_ in relation to cognition. In Supplementary Tables S1 and S2, the correlation coefficients between DDT_V S_ or DTT_V N_ and a particular cognitive test are always almost identical. For correlation differences of this size to reach significance, at least 250 but often several 1000 participants would need to be tested. Similarly, in the CCA the weighting of the root factor, that is the effect of hearing and cognition, is very similar for the two types of digit triplet test (0.20 and 0.32). Lastly, in the stepwise regression analyses reported in **Table 3** both types of digit triplet test showed the same predictive pattern for hearing (yes) and cognition (no). Hence, we conclude that there were no distinguishing features in these analyses to suggest that the relationship with cognition differs between DTT_V S_ and DTT_V N_.

### Aim 2: Assessing the Relationship between Speech Perception Performance and Self-Reported Outcomes

#### Prediction 2.1. Hearing-Specific Questionnaires will Demonstrate a Greater Association with Speech Perception Performance than Generic Health Measures

##### Correlational analyses

Simple Pearson product-moment correlations for the association between self-report measures and the four speech perception tests are shown in **Table 4** The results show that the general HRQoL questions (general EQ5-5D) were not correlated with performance on any of the speech perception tests. In contrast, hearing and communication-specific measures (hearing-specific EQ-5D, ALDQ, GHABP, and SSQ) were significantly associated with some, but not all, speech perception tests. Hence, only questionnaires that assessed hearing-related aspects of self-report correlated with behavioral measures of speech perception.

**Table 4:** Pearson product-moment correlations between each of four speech perception tests and self-report questionnaires.

		Correlation				Diff. significant			
		PD	DTT_V S_	DTT_V N_	ASL	DTT_V S_	DTT_V N_	ASL
Health-related quality of life (HRQoL)	EQ-5D	General	0.10	0.11	0.08	0.17	DTT_V N_^a^ ASL^b^	DTT_V S_^a^	DTT_V S_^b^
		N	41	42	42	42			
		Hearing-specific	0.10	0.26	0.38^∗^	0.42^∗∗^	PD^b^	PD^b^	PD^b^
		N	41	42	42	42			
Self-report of hearing	ALDQ		0.25	0.25	0.35^∗^	0.26			
		N	43	44	44	44			
	GHABP		0.04	0.26	0.33^∗^	0.02	ASL^a^	PD^b^, ASL^b^	DTT_V S_^a^ DTT_V N_^b^
		N	43	44	44	44			
	SSQ		0.06	0.37^∗^	0.29^∗^	0.25	PD^b^		
		N	43	44	44	44			

*Acronyms as for **Table 1** Significant two-tailed correlations are shaded. ^∗^*p* < 0.05, ^∗∗^p < 0.01, ^a^p_(one-sided)_< 0.05, ^b^p_(two-sided)_< 0.05*.

#### Prediction 2.2. Correlations with Speech Perception Performance will be Largest for Questionnaires that Capture Aspects of Listening Important for that Particular Speech Perception Test

##### Correlational analyses – differences between tests

**Table 4** also shows that DTT_V N_ had the greatest number of significant correlations with self-report questionnaires, in particular with the hearing-specific EQ-5D and the hearing-specific questionnaires (ALDQ, GHABP, and SSQ). In contrast, Phoneme discrimination was not correlated with any self-report questionnaires. Both DTT_V S_ and Sentence perception were only each correlated with one self-report scale (SSQ and hearing-specific EQ-5D, respectively). A direct comparison of correlation sizes between speech perception and self-report measures (‘Diff significant’) showed that even though DTT_V N_ had numerous significant correlations with self-report measures, the coefficients were not significantly greater than those for the ASL or DTT_V S_, except for ASL in the case of GHABP. Hence, it is not clear whether one particular SiN test captures self-report significantly better than other speech perception tests.

##### Canonical correlational analyses

The four speech perception tests were entered as one set of variables, while the hearing-specific EQ-5D, the ALDQ, GHABP, and SSQ were entered as the other set. The overall multivariate model, based on 41 cases, indicated that there was evidence for an overall relationship between the two sets of variables (Wilks’ lambda, *p* = 0.005). Univariate regression analyses within the CCA model indicated that only performance on Phoneme discrimination was not significantly related to self-report, while performance on all other speech perception tests was significantly related to self-report (DTT_V S_: *p* = 0.016; DTT_V N_: *p* = 0.005; ASL: *p* = 0.025). The first canonical root explained 38% of shared variance, the second 26%, the third 10%, and the fourth 9%, with only the first two roots being significant [all canonical correlations included: *F*(16,101) = 2.37, *p* = 0.005; first root removed: *F*(9,83) = 2.09, *p* = 0.04]. The correlations and canonical coefficients for the significant root solutions 1 and 2 are shown in Supplementary Table S4. Examination of the loadings suggests a picture similar to that presented by the correlations reported in **Table 4** The first canonical root suggests that lower scores on hearing-specific EQ-5D and higher (i.e., richer) scores on self-rated sound environments are related to higher SNR in the DTT_V N_. This replicates the negative correlation between hearing-specific EQ-5D and DTT_V N,_ and the positive correlation between ALDQ and DTT_V N_. The second canonical root suggests that better self-rated activity and participation scores are related to lower SNRs in the DTT_V N_. This replicates the negative correlation between GHABP and DTT_V N_.

DTT_V N_ showed the richest pattern of correlations with self-report questionnaires, although this difference in pattern was to some extent difficult to establish in terms of significant differences in correlation size. This difference in association between speech perception tests and questionnaires was also reflected in the canonical correlations. Despite differences being small, the overall pattern of results nevertheless suggests that speech perception tests differ in how closely their performance is associated with aspects of self-reported hearing, and that performance on the DTT_V N_ showed the closest correspondence with all the hearing-related self-report scales.

#### Prediction 2.3. Procedural Differences in Identifying SNR for Speech Perception Performance may Lead to Different Associations with Self-report Scales. In Particular, Increasing the Level of Background Noise to Reduce Perceptual Accuracy may be Uniquely Associated with Functioning in Challenging Auditory Environments

This hypothesis is assessed by comparing the differences in correlation between self-report scales and DTT_V S_ or DTT_VN_, respectively. **Table 4** shows that the differences in correlation between self-report scales and the two speech perception tests are small. For the hearing-specific EQ-5D, ALDQ, and GHABP the differences are 0.12, 0.10, and 0.08 which equates to a small effect. In the context of this study more than 80 participants would be required for an effect of this magnitude to reach significance. Nevertheless, the canonical correlations suggest the involvement of particularly DTT_V N_ in several correlations of different aspects of the speech perception.

## Discussion

Listening can be assessed behaviorally with speech perception tests or subjectively with self-report measures. Which measure is chosen to assess an outcome, either in clinical or research evaluations, depends on many factors including availability, familiarity, and popularity of a particular measure. Less consideration might be given to either the specific aspect of listening that is assessed by a particular test or questionnaire, or the contribution of cognitive functioning to speech perception performance. This investigation considered these relationships to help inform outcome selection for clinical and research purposes.

We assessed the relationship between measures of speech perception and hearing, cognition, and self-reported outcomes. Speech perception tests varied in complexity from low (phonemes in quiet) to medium (words in steady-state speech-shaped noise) to high (sentences in 8 Hz modulated noise). Cognitive tests either emphasized the storage and processing of information (WM), or attention and cognitive control. Information storage and processing capacities were measured with digit span tasks (forward and backward) and a VLM task, while attention and cognitive control was measured by means of focused and divided attention tasks. We also assessed the effect of the protocol for changing the SNR in one of the speech tasks (Digit Triplet Test) by varying either the speech or the noise. This allowed us to assess whether the procedure affected either the extent of cognitive contributions to the speech task, or the extent to which speech perception performance correlated with self-reported aspects of hearing. In the following, each hypothesis and associated results is considered in turn.

### Assessing the Relationship between Speech Perception Performance and Cognition

#### Prediction 1.1. Speech Perception Performance will be Associated with Cognition, and this will be Moderated by Hearing Sensitivity

Initial correlation analyses showed some correlations between speech perception and cognitive performance. This pattern remained largely unchanged even when hearing loss was taken into account, despite the fact that hearing loss had a significant influence on the speech perception results. Age did not independently contribute to the speech perception results, possibly because the age range of the participants was restricted (50–74 years).

The influence of hearing loss on speech perception is well documented in the literature (e.g., Humes and Roberts, 1990; van Rooij and Plomp, 1990; Humes and Dubno, 2010) and the results of this study fit within this body of evidence. That cognition also presented as a considerable factor for speech perception performance in some tests, above and beyond hearing loss, is also in accordance with previous results (e.g., Akeroyd, 2008; Houtgast and Festen, 2008; Humes et al., 2013). Finally, studies have also previously shown that the contribution of hearing and cognition to speech perception performance varies depending on the background in which the speech task is presented, with adverse noise conditions more likely to invoke cognitive processes than listening in quiet (e.g., van Rooij and Plomp, 1990; Wingfield et al., 2005; Rönnberg et al., 2010). However, the complexity of a listening situation can vary in more ways than just the presence of absence of background noise. Thus, the second prediction was investigated to assess how the contribution of cognition changed depending on the listening situation.

#### Prediction 1.2. The Contribution of Cognition will Increase as the Complexity of the Speech Perception Task Increases

The complexity of the listening situation in the current study is determined by (i) the target speech, which comprised phonemes, words or sentences, (ii) the background, which was steady-state and 8-Hz modulated noise, and (iii) the listening task itself, which included recognition and comprehension. How these different aspects of the listening situation affect the relationship between cognitive processing and speech perception have so far inspired surprisingly little systematic research, apart from the general demonstration that correlations with cognitive processes are greater when listening to speech in adverse noise conditions than when listening in quiet (e.g., van Rooij and Plomp, 1990; Wingfield et al., 2005; Rönnberg et al., 2010). This study took a first step toward understanding if and how the contribution of cognitive components differed for various SiN conditions, and whether this depended on the exact pairing of cognitive subcomponent and complexity of listening situation.

The choice of cognitive subcomponents to be assessed was informed by previous work that had clearly demonstrated a role of WM for SiN perception (see Akeroyd, 2008 for a review). However, WM tests differ in respect to the emphasis they give to different subcomponents of cognition (storage, processing, inhibition, cognitive control) depending on the model they are based on. The current study tested all of these subcomponents. On a general note, the study showed that correlations between cognitive components and speech perception occurred mainly for the most complex speech perception test (sentences in 8 Hz modulated noise), while digit perception in steady-state noise showed only few correlations, and phoneme discrimination in quiet showed none. This result was also borne out in the hierarchical regression analyses where only performance on the sentence perception task was reliably associated with cognition.

Distinct cognitive profiles for different speech perception tests emerged, in particular for the sentence perception and DTT_V S_. Supplementary Table S2 shows that not only performance on the NVIQ and focused attention tasks correlated significantly with sentence perception but also that this correlation was significantly higher than those for the same tests with DTT_V S_. For the divided attention decrement, the situation was reversed in that this test only showed a significant correlation with DTT_V S,_ which was significantly higher than with sentence perception. At this point we can only speculate why this might have happened as we did not systematically manipulate aspects of the listening task to assess whether it was the change in target speech (from digits to sentences) or the change in background noise (from steady-state to modulated noise) that led to this change in correlation profile. It may be the correlation between sentence perception and digit span occurred because the successful repetition of a sentence involved significant WM storage. It is also possible that focused attention on the words within a sentence was particularly beneficial because perception of words may result in successful inference of the rest of the sentence, whereas such an inference would not be possible for strings of single digits. Conversely, for digit triplet in noise, maybe successful listening meant being able to tolerate both signals, the digits and noise, rather than trying (and failing) to ignore the noise, and listeners who were best able to do this also had the smallest divided task decrement.

These data offer some initial suggestions that may help to reconcile the inconsistencies existing in the literature on the relationship between cognition and speech perception, and may thereby help to increase our understanding of the exact relationship between speech perception and cognition. The results suggest that the relationship between speech and cognition can be specific to the tests used, and thus simply referring to speech perception and cognition may ignore important distinctions. Being more specific about cognition and speech may help us understand why the reading span task, as a complex WM measure, correlates with speech perception when measured with sentences in noise (Desjardins and Doherty, 2013; Moradi et al., 2014) but not when measured with syllables (Kempe et al., 2012). Similarly, performance on the VLM task may predict performance on a particular word perception task (Gatehouse et al., 2003) but not on a sentence perception task (Rudner et al., 2008).

Lastly, when assessing the effect of WM and attention for cognition (Att) separately by means of latent principal component factors, it was the attention and NVIQ, rather than WM that were associated most closely with sentence perception performance. This result contrasts with previous studies which have shown a clear correlation between WM and SiN perception in older listeners (Humes et al., 2006; Rudner et al., 2008).

#### Prediction 1.3. Where Procedural Differences in Identifying SNR Occur while the Speech and Background Signals are Identical, we Expect Comparable Associations with Cognition if these Associations are Driven by Signal Complexities and not Procedural Differences

An interesting dichotomy of results emerged: ASL and DTT_V S_ which both changed SNR in the same way (constant noise level and adjusted speech) but used different speech material (sentences and words) showed statistically reliable differences in their cognitive profiles (i.e., their correlations with specific cognitive tests). Conversely, DTT_V S_ and DTT_V N_, which both used different methods to adjust SNR, but also used the same speech material and background sounds, showed similar cognitive profiles. It might be argued that the similarity in results between DTT_V S_ and DTT_V N_ was due to insufficient power rather than the true absence of an effect. However, the significant differences between ASL and DTT_V S_ showed that the effects in the data were strong enough to show significant differences when they existed. Moreover, power analyses based on the current effect sizes showed that for most profile differences several 100 data points would have been needed to show significant differences. Therefore we conclude that our results were consistent with the prediction, and that both methods of setting SNRs place similar cognitive demands on the listener and are equally suited for setting SNR if cognitive demand is the main concern.

### Assessing the Relationship between Speech Perception Performance and Self-Reported Outcomes

#### Prediction 2.1. Hearing-Specific Questionnaires will Demonstrate a Greater Association with Speech Perception Performance than Generic Health Measures

Questionnaires that assess activity and participation relating to hearing and communication correlated more highly with speech perception outcomes than general HRQoL questionnaires. These results are consistent with other studies (Joore et al., 2002; Stark and Hickson, 2004; Chisolm et al., 2007) and this prediction.

#### Prediction 2.2. Correlations with Speech Perception Performance will be Largest for Questionnaires that Capture Aspects of Listening Important for that Particular Speech Perception Test

Similar to the cognitive results, different patterns of correlation also existed between self-report measures and speech perception tests. Phoneme discrimination correlated least with self-report measures. At this point we cannot say whether this result occurred because of the low complexity of the speech material or the lack of background noise, or indeed both. All other speech perception tests showed correlations with at least one self-report outcome. Although DTT_V N_ showed the richest pattern of significant correlations with self-report measures, the differences in correlation to the other speech perception tests involving at least words or sentences only became significant for one questionnaire (i.e., GHABP), and only in contrast to one speech perception test (i.e., ASL). In summary, these results would suggest that these speech perception tests all measure similar aspects of self-reported experiences but that these aspects are represented most strongly in the DTT_V N_.

#### Prediction 2.3. Procedural Differences in Identifying SNR for Speech Perception Performance may Lead to Different Associations with Self-Report Scales. In Particular, Increasing the Level of Background Noise to Reduce Perceptual Accuracy may be Uniquely Associated with Functioning in Challenging Auditory Environments

One particularly interesting aspect of the study was the administration of the same speech task, the DTT, with two different administration protocols and the resulting changes in the correlation with self-reported outcomes. The results showed that administering the task with variable noise (DTT_V N_) was significantly associated with aspects of communication (hearing-specific EQ-5D), ALDQ, communication (GHABP), and SSQ. However, administering the task with variable speech (DTT_V S_) was only significantly associated with the SSQ. Moreover, in the CCA, DTT_V N_ contributed substantially more to the first and second canonical root than DTT_V S,_ suggesting that DTT_V N_ is more likely to play a prominent role in hearing and communication functions. This is relevant to the way in which the DTT was administered, and highlights the fact that practitioners and researchers alike should think about their question of interest before deciding for a particular test. If aspects of speech perception are of most interest then fixing the noise level and varying the speech appears most effective. However, if aspects of communication and participation restriction of the listening experience are of interest, then choosing to keep the level of the speech constant and varying the noise might be more appropriate. These results are also interesting in the light of previous research, where some studies have used variable speech (Plomp and Mimpen, 1979; Smits et al., 2004; George et al., 2006; Jansen et al., 2010; Vlaming et al., 2011), while others have used variable noise (Mayo et al., 1997; Rogers et al., 2006), with one study even using both methods in the same experiment (Smits et al., 2013). If communication ability and noise tolerance beyond intelligibility is a consideration then researchers need to choose deliberately between the two SNR methods.

### Limitations

There are a number of limitations to this investigation. First, this study was designed as an auditory training intervention trial. Therefore the measures were included for the purpose of assessing the intervention, and not specifically selected for the purposes of the current evaluation. As such, speech and cognitive outcomes were limited to the outcomes of that study, and were not chosen specifically to represent a fully factorial combination of the complexities of target speech and background noise. Instead they were meant to sample broadly across the continuum of listening situations with varying complexities in foreground and background simultaneously. As a result, changes in correlations between cognitive function and speech perception cannot be unambiguously attributed to changes in the complexity of the target speech. Future purpose-designed studies will enable a finer-grained analysis of the issues raised in this investigation and investigate in greater detail the complexity of the foreground and background signal to listening demands.

Another consequence of the intervention trial design is the fact that the number of participants (*n* = 44), while large for a training study, is rather small for the type of analyses performed here. This limits the power and generalizability of the results. The coarse differentiation of speech perception test complexity and the relatively small number of participants makes this study strictly exploratory.

Third, the inherent nature of a speech perception test dictates that the speech content is unlikely to be highly relevant to the individual, nor particularly interesting. This may therefore impact on an individual’s motivations to pay attention and actively listen to the speech content (see Henshaw et al., in press for an overview).

Fourth and finally, the participants in this study were adults with mild SNHL who did not wear hearing aids. Thus, this investigation adds to research on the relationship between cognition and self-report measures to different speech perception tests in un-aided listening (Cox and Alexander, 1992; Humes et al., 2013). This stand-alone examination cannot tell us how these relationships may change once hearing intervention occurs, e.g., once hearing aids are fitted.

## Conclusion

The results of this study show that different speech perception tests engage cognition to different extents, and reflect different subjective aspects of the self-reported listening experience. These results suggest that practitioners and researchers should think carefully about the objective outcome measures they choose as different speech and cognitive tests will highlight different aspects of listening and engage different cognitive processes. One way in which this could be useful for audiological practice is to choose a speech perception test that highlights those aspects of communication and participation that the patient indicated as being important and/or difficult for them. Alternatively, tests could be specifically chosen to maximize or minimize cognitive influences, which might put a listener at an advantage or a disadvantage. Finally, to assess change in speech perception performance as a result of an intervention, researchers or clinicians should select speech perception tests that are associated with the intended mechanism of benefit of that intervention in order to adequately detect any associated change in performance (see Ferguson and Henshaw, 2015).

## Author Contributions

MF designed the study. AH analyzed and interpreted the data. AH wrote the manuscript. AH, MF, and HH contributed to critical discussions. AH and MF revised the manuscript. All authors approved the final version of the manuscript for publication. All authors agree to be accountable for all aspects of the work and in ensuring that questions related to the accuracy or integrity of any part of the work are appropriately investigated and resolved.

## Conflict of Interest Statement

The authors declare that the research was conducted in the absence of any commercial or financial relationships that could be construed as a potential conflict of interest.
