# Supplementary material for: The relationship of speech intelligibility with hearing sensitivity, cognition, and perceived hearing difficulties varies for different speech perception tests
Source: Front Psychol. 2015 Jun 16;6:782. doi: 10.3389/fpsyg.2015.00782 (PMC4468362; doi:10.3389/fpsyg.2015.00782)
Supplement: Supplementary file 1 [file Data_Sheet_1.PDF]

frontiers

Supplementary Material

The relationship of speech intelligibility with hearing sensitivity, cognition, and perceived hearing difficulties varies for different speech perception tests

Antje Heinrich[1]*, Helen Henshaw[2], Melanie A. Ferguson[2,3]
[1]Medical Research Council Institute of Hearing Research, Nottingham, UK, [2]National Institute for Health Research-Nottingham Hearing Biomedical Research Unit, Otology and Hearing Group, Division of Clinical Neuroscience, School of Medicine, University of Nottingham, Nottingham, UK, [3]Nottingham University Hospitals NHS Trust, Nottingham, UK

*** Correspondence:** Dr Antje Heinrich, MRC Institute of Hearing Research, University Park, Nottingham, NG7 2RD, UK
antje.heinrich@ihr.ac.uk

1. Supplementary Data

To provide the reader with full results, Table 1 reports bivariate correlations between all speech and cognitive tests. As three of the four speech tests demonstrated significant correlations with hearing (BEA), Table 2 provides the same correlation correlations with hearing thresholds partialled out. Both tables demonstrate a consistent pattern of results, with only sentence perception and DTT_{VS} showing significant correlations with cognitive tests. In particular, two of the significant correlations between cognitive tests and sentence perception (Matrix Reasoning, TEA6) are significantly higher than for DTT_{VS}, in the case of nonverbal IQ (Matrix Reasoning) exclusively so. Conversely, the significant correlation of DTT_{VS} with the dual task decrement (DTD) was significant higher only than the correlation of DTD with sentence perception.

Supplementary Table 1. Simple Pearson product-moment correlations between each of four speech tests, age, hearing and cognitive tests. PD = Phoneme discrimination, Digit Triplet Test with variable speech (DTT_{VS}) or variable noise (DTT_{VN}), ASL = Adaptive Sentence List. BEA = better ear $average_{(0.5\text{-}4kHz)}$, NVIQ = nonverbal intelligence quotient, VLM = visual letter monitoring, RT=reaction time, TEA=Test of Everyday Attention, DTD (dual-task decrement). Significant correlations are shaded. †$p \leq 0.07$, *$p < 0.05$, **$p < 0.01$. 'Diff significant' denotes speech tests for which the correlation with a particular cognitive test differed reliably based on their z-values ($p_{(one\text{-}sided)} < 0.05$). Typically, a difference in correlation of approximately r = 0.3 was required for the change to become statistically significant.

				Speech tests				Diff significant	
				PD	DTT_{VS}	DTT_{VN}	ASL	DTT_{VS}	ASL
Age		r		.17	.15	.07	.19		
		N		43	44	44	44		
Hearing		BEA $_{(0.5\text{-}4kHz)}$		.28	.49**	.44**	.39**		
		N		43	44	44	44		
Cognition	NVIQ	Matrix Reasoning		-.12	-.02	-.07	-.27	ASL	DTT_{VS}
		N		43	44	44	44		
	Working memory	Digit span	Forward	-.12	-.07	-.09	-.20		
			N	43	44	44	44		
			Backward	-.19	-.13	.-14	-.32*		
			N	43	44	44	44		
		VLM	Accuracy (Hits)	-.09	-.13	-.10	.02		
			N	39	40	40	40		
			Speed (RT) ms	-.09	.00	.00	-.15		
			N	39	40	40	40		
	Attention	TEA	Subtest 6	.00	-.04	-.05	-.38**	ASL	PD DTT_{VS} DTT_{VN}
			N	42	43	43	43		
			Subtest 7	-.10	-.26	-.22	-.29†		
			N	42	43	43	43		
			DTD	.12	.29†	.24	.08		
			N	42	43	43	43		

Supplementary Table 2. Pearson product-moment correlations between the four speech tests and cognitive tests with hearing (BEA) partialled out. Acronyms as Table 1. Significant correlations ($p<.05$) are shaded. †$p \leq 0.07$, *$p < 0.05$, **$p < 0.01$. ‘Diff significant’ denotes speech tests for which the correlation with a particular cognitive test differed reliably based on their z-values ($p_{(one\text{-}sided)} < 0.05$). Typically, a difference in correlation of approximately $r = 0.3$ was required for the change to become statistically significant.

				Correlation				Diff significant	
				PD	DTT$_{VS}$	DTT$_{VN}$	ASL	DTT$_{VS}$	ASL
Cognition	NVIQ	Matrix Reasoning		-.14	-.06	-.11	-.32*	ASL	DTT$_{VS}$
		N		40	41	41	41		
	Working memory	Digit span	Forward	-.13	-.09	-.11	-.23		
			N	40	41	41	41		
			Backward	-.16	-.08	-.09	-.29†		
			N	40	41	41	41		
		VMT	Accuracy (Hits)	-.03	-.03	.00	.12		
			N	36	37	37	37		
			Speed (RT)	-.03	.11	.11	-.08		
			N	36	37	37	37		
	Attention	TEA	Subtest 6	.04	.05	.02	-.36*	ASL	PD DTT$_{VS}$ DTT$_{VN}$
			N	39	40	40	40		
			Subtest 7	-.08	-.26	-.21	-.29†		
			N	39	40	40	40		
			DTD	.13	.35*	.27	.09	ASL	DTT$_{VS}$
			N	39	40	40	40		

Supplementary Table 3. Correlation and standardized canonical coefficients between BEA / cognition (single latent factor "Cogn") and the four speech tests.

	First canonical correlation		Second canonical correlation	
	Correlation	Coefficient (weight)	Correlation	Coefficient (weight)
BEA	.90	.84	-.44	-.55
Cognition (Cogn)	-.55	-.44	-.84	-.91
PD	.57	.21	-.01	.03
DTT_{VS}	.89	.20	-.36	-.65
DTT_{VN}	.82	.32	-.45	-.55
ASL	.88	.50	.45	1.15

Supplementary Table 4. Correlation and standardized canonical coefficients between self-report variables and the four speech tests.

	First canonical correlation		Second canonical correlation	
	Correlation	Coefficient (weight)	Correlation	Coefficient (weight)
Q6-10	-.80	-.63	-.41	-.56
ALDQ	.60	.53	-.27	-.33
GHABP	-.50	-.28	.49	1.03
SSQ	-.39	-.10	-.26	-.68
PD	.10	-.12	-.08	-.49
DTT_{VS}	.64	-.40	.15	.68
DTT_{VN}	.93	1.06	-.20	-1.14
ASL	.68	.41	.63	1.00
